# Atypical NMDA receptor expression in a diffuse astrocytoma, MYB- or MYBL1-altered as a trigger for autoimmune encephalitis

**DOI:** 10.1007/s00401-022-02447-y

**Published:** 2022-06-21

**Authors:** Marc Nikolaus, Arend Koch, Werner Stenzel, Sefer Elezkurtaj, Felix Sahm, Anna Tietze, Laura Stöffler, Jakob Kreye, Pablo Hernáiz Driever, Ulrich W. Thomale, Angela M. Kaindl, Markus Schuelke, Ellen Knierim

**Affiliations:** 1 Department of Neuropediatrics, Charité-Universitätsmedizin Berlin, Corporate Member of Freie Universität Berlin, Humboldt-Universität zu Berlin, and Berlin Institute of Health (BIH), Augustenburger Platz 1, Mittelallee 8, 13353 Berlin, Germany; 2 Center for Chronically Sick Children, Charité-Universitätsmedizin Berlin, Corporate Member of Freie Universität Berlin, Humboldt-Universität zu Berlin, and Berlin Institute of Health (BIH), Berlin, Germany; 3 Berlin Institute of Health (BIH), Charité-Universitätsmedizin Berlin, Corporate Member of Freie Universität Berlin, Humboldt-Universität zu Berlin, and Berlin Institute of Health (BIH), Berlin, Germany; 4 Department of Neuropathology, Charité-Universitätsmedizin Berlin, Corporate Member of Freie Universität Berlin, Humboldt-Universität zu Berlin, and Berlin Institute of Health (BIH), Berlin, Germany; 5 Institute of Pathology, Charité-Universitätsmedizin Berlin, Corporate Member of Freie Universität Berlin, Humboldt-Universität zu Berlin, and Berlin Institute of Health (BIH), Berlin, Germany; 6 Institute of Neuroradiology, Charité-Universitätsmedizin Berlin, Corporate Member of Freie Universität Berlin, Humboldt-Universität zu Berlin, and Berlin Institute of Health (BIH), Berlin, Germany; 7 Department of Neurology and Experimental Neurology, Charité-Universitätsmedizin Berlin, Corporate Member of Freie Universität Berlin, Humboldt-Universität zu Berlin, and Berlin Institute of Health (BIH), Berlin, Germany; 8 Department of Pediatric Oncology/Hematology and German HIT-LOGGIC-Registry for Low-Grade Glioma in Children and Adolescents, Charité-Universitätsmedizin Berlin, Corporate Member of Freie Universität Berlin, Humboldt-Universität zu Berlin, and Berlin Institute of Health (BIH), Berlin, Germany; 9 Department of Pediatric Neurosurgery, Charité-Universitätsmedizin Berlin, Corporate Member of Freie Universität Berlin, Humboldt-Universität zu Berlin, and Berlin Institute of Health (BIH), Berlin, Germany; 10 Institute of Cell Biology and Neurobiology, Charité-Universitätsmedizin Berlin, Corporate Member of Freie Universität Berlin, Humboldt-Universität zu Berlin, and Berlin Institute of Health (BIH), Berlin, Germany; 11 NeuroCure Cluster of Excellence, Charité-Universitätsmedizin Berlin, Corporate Member of Freie Universität Berlin, Humboldt-Universität zu Berlin, and Berlin Institute of Health (BIH), Berlin, Germany; 12 Department of Neuropathology, Ruprecht-Karls-University Heidelberg, Clinical Cooperation Unit Neuropathology, German Cancer Research Center (DKFZ), Heidelberg, Germany; 13 German Center for Neurodegenerative Diseases (DZNE), Berlin, Germany

Triggers of anti-N-methyl-D-aspartate receptor encephalitis (NMDARE) include *Herpes simplex* encephalitis (HSE) [11] and ovarian teratomas [14]. The latter occur in 50% of adult NMDARE patients and are distinct from ovarian teratomas not associated with encephalitis. They contain neuronal tissue more often and exhibit (ganglio)glioma-like features with dysmorphic neurons and atypical NMDAR expression [4, 7]. Ectopic NMDARs could, therefore, initiate or maintain the formation of anti-NR1 antibodies [8]—an anti-tumor response leading to autoimmune encephalitis. Interestingly, brain tumors have not been implicated as triggers of NMDARE [2], despite their prevalence and expression profile that includes NMDARs [12].

Here, we present data on atypical NMDAR expression in glioneuronal brain tumors, in which dysmorphic neurons might induce antibody formation leading to autoimmune encephalitis.

We followed an atypical refractory NMDARE in a 21-month-old girl for more than 2 years. After a 4-week history of gait disturbance, behavioral changes, and seizures, MRI showed a T_2_-hyperintensity in the cerebellar white matter, which was interpreted as inflammatory lesion (Fig. 1d). CSF analysis revealed pleocytosis, type 2 oligoclonal bands, and intrathecal antibody synthesis. Screening for anti-neuronal antibodies detected anti-NR1 IgG in CSF (1:1,000) and serum (> 1:10,000) confirming NMDARE (Fig. 1a–c). With intensive immunotherapy, the child’s condition initially improved but a protracted course developed with fluctuating antibody titers and relapses refractory to therapy (Fig. 1g). When subsequent imaging showed growth of the presumed inflammatory lesion, navigated needle biopsy of the now suspected cerebellar tumor was performed (Fig. 1e). Based on histomorphology and molecular profile, we detected a diffuse astrocytoma, *MYB* or *MYBL1*-altered with *MYBL1:MMP16*-fusion [15] (Fig. 1i–o). In contrast to the *MYB/MYBL1*-altered astrocytoma presented here, these tumors are usually not located in the cerebellum and do not contain dysmorphic neurons [5]. After a third recurrence, the patient underwent subtotal tumor resection (Fig. 1f). This resulted in a sharp decrease in anti-NR1 titer (1:3) and significant sustained clinical improvement (Fig. 1g, h, detailed case in Supplementary 1, online resources).

**Fig. 1:**
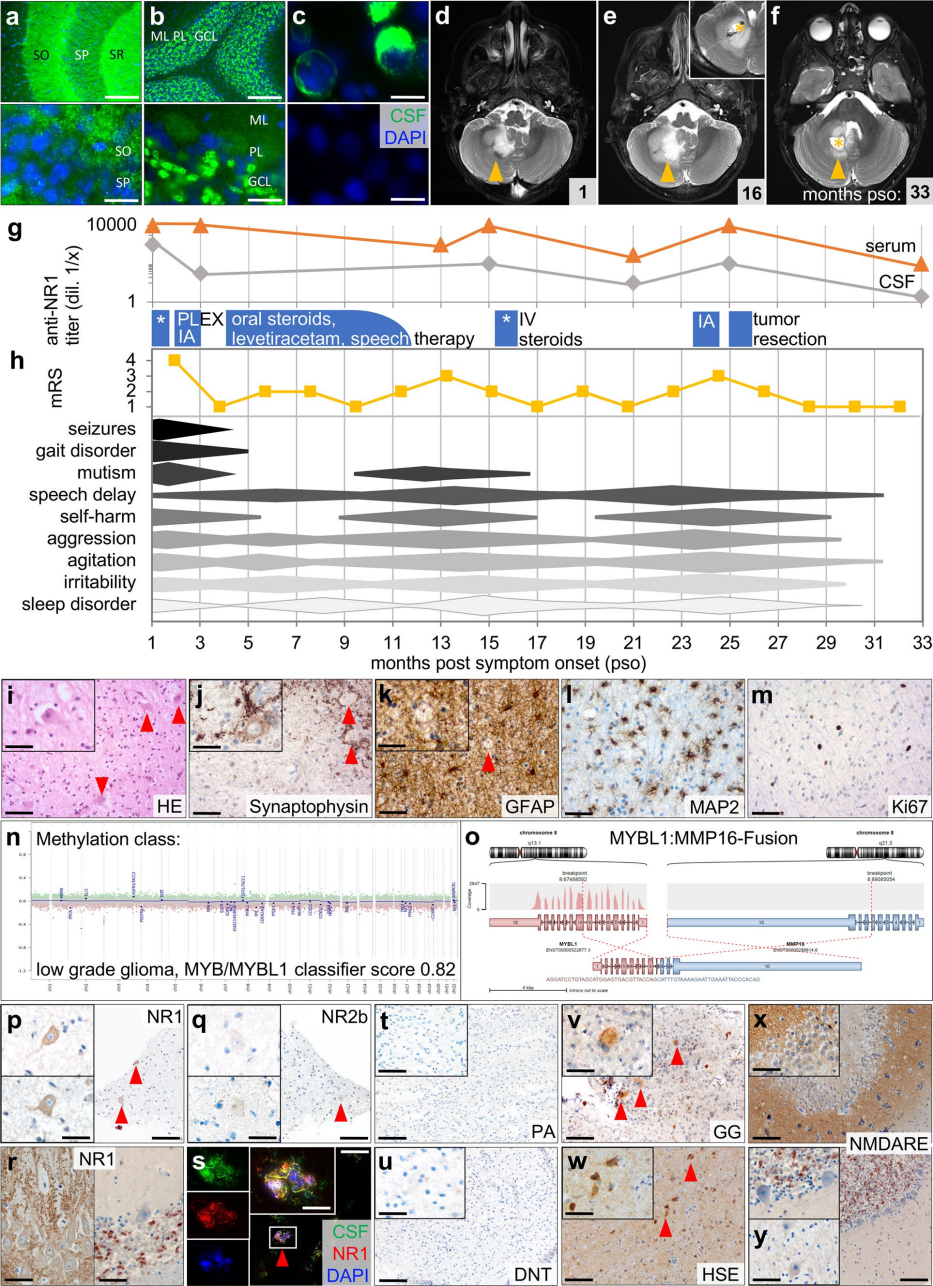
Atypical NMDAR expression in dysmorphic neurons of diffuse astrocytoma, MYB/MYBL1-altered, detected in a patient with NMDAR encephalitis. Immunostaining with CSF shows typical anti-NMDAR neuropil signal (green, DAPI co-localization blue) in mouse hippocampus (**a**) and cerebellum (**b**), and binding to NR1-expressing HEK293T-cells (negative control below) (**c**). SO: *Stratum oriens*, SP: *Stratum pyramidale*, SR: *Stratum radiatum*, ML: molecular layer, PL: Purkinje cell layer, GCL: granular cell layer. Size bars: 100 µm **(**top **a**, **b)**, 20 µm **(**bottom **a**, **b**; both **c**). MRI 1 month post-symptom onset (pso) shows white matter T_2_-hyperintensity in right cerebellar hemisphere with minimal contrast uptake (**d**). Navigated biopsy (inset) of the growing mass 16 months pso allows tumor diagnosis (**e**). Follow-up 6 months after subtotal resection shows decrease in residual mass (**f**). Arrows indicate tumor; asterisks indicate biopsy and resection sites. Patient’s CSF and serum anti-NR1 titers fluctuate during treatment with steroids, plasmapheresis (PLEX), and immunoadsorption (IA), and drop after surgery (**g**). Symptoms, progression and treatment response assessed by physical examination and modified Rankin Scale (mRS) are presented semiquantitatively (**h**). Histomorphologic characterization reveals moderately cell-rich isomorphic glial tumor with narrow, pale eosinophilic cytoplasm and fibrillary processes. Dysmorphic neurons are seen within glial tumor matrix (**i**). Dysmorphic neurons located within tumor tissue are labeled with antibodies against synaptophysin (**j**). Tissue shows diffuse matrix-related GFAP reaction (**k**). Glial tumor cells with fibrillary processes are highlighted in MAP2 staining (**l**). There is little proliferative activity (Ki67-labeling index 3%) (**m**). On methylation analysis (EPIC), tumor is assigned to “low grade glioma, MYB/MYBL1” (classifier score v11b4 0.82 and v12.5 0.93). There is a flat CNV profile with no significant chromosomal loss or gain (**n**). RNA sequencing demonstrates MYBL1:MMP16 fusion (**o**). Dysmorphic neurons (arrowheads) in patient’s tumor show NMDAR-positivity with atypical concentration in somata rather than in neuropil (**p**). Signal is NR1-specific (**q**). Hippocampus (left) and cerebellum of healthy controls show typical neuropil signal (**r**). Immunostaining of fresh–frozen patient tumor with patient CSF (green) and commercial anti-NR1 (red) overlaps (yellow) confirming atypical NMDAR expression (**s**). Size bars: 200 µm (**p**, **q**, **s**), 100 µm (**r**), 20 µm (insets). Immunostaining with tumors of different glial and neuronal composition from individuals without autoimmune encephalitis show no NR1 signal in *pilocytic astrocytoma* (PA) (**t**), weak neuropil staining without NMDAR-positive somata in *dysembryoplastic neuroepithelial tumor* (DNT) (**u**), and neuropil staining with atypical NMDAR expression on somata of dysmorphic neurons (arrowheads) in *ganglioglioma* (GG) (**v**). Similar NR1 signal is seen in CNS tissue from patients with *Herpes simplex* encephalitis (HSE), where areas of inflammatory infiltration show reduced neuropil staining but neurons with NMDAR-positive somata (**w**). NMDARE without brain tumor or viral infection shows normal NMDAR pattern in hippocampus (**x**, top**)**, cerebellum (bottom), and dentate nucleus (**y**). Size bar: 200 µm (insets 100 µm)

In contrast to this case and unlike adults, the clinical presentation of NMDARE in children is dominated by neurologic rather than psychiatric symptoms, associated tumors are less common, and complete recovery is more likely [14]. In most children, no triggers are found.

To investigate a possible association between NMDARE and brain tumors, we analyzed tumor samples from the index patient (methods in Supplementary 2, online resources). Immunohistochemistry revealed atypical NR1-specific NMDAR positivity in somata of dysmorphic neurons detectable in the tumor (Fig. 1p, q). Glial tumor areas were negative. In contrast to cortical, hippocampal, and cerebellar control tissue, no neuropil signal was seen (Fig. 1r). Immunostaining with patient CSF was consistent with anti-NR1 signal of commercial antibodies. This confirmed atypical NMDAR expression on the patient’s dysmorphic neurons and suggested that autoantibodies binding the tumor were also responsible for NMDARE (Fig. 1s). Atypical NMDAR expression was specific for neuronal tissue. When analyzing low-grade tumors with different glial and neuronal composition from patients without reported autoimmune encephalitis, e.g., *pilocytic astrocytoma*, *dysembryoplastic neuroepithelial tumor*, or *ganglioglioma*, atypical NMDAR expression was detected only on dysmorphic neurons in *ganglioglioma*—and in the aforementioned NMDARE-associated ovarian teratomas (Fig. 1v, Supplementary Fig. 3 and 4, online resources). Tumors without such neurons were negative (Fig. 1t, u). However, we found similar atypical NMDAR expression within inflamed CNS tissue of HSE patients. Areas of severe inflammatory infiltration showed reduced neuropil signal, whereas NMDAR was expressed in the somata of neurons (Fig. 1w). Tissue from NMDARE patients without brain tumor or infection showed normal neuropil staining (Fig. 1x, y), making an epiphenomenon unlikely. Rather, atypical NMDAR expression is either a response to anti-tumor immune reactions or property of the dysmorphic neuronal tissue that facilitates such an anti-NMDAR antibody-mediated response.

Based on this example, it cannot be clarified with certainty to what extent dysmorphic cells present in tumor tissue or local neurons, e.g., from deep cerebellar nuclei, altered by inflammatory processes may induce NMDARE. The latter usually do not show atypical NMDAR expression (Supplementary Fig. 5, online resources).

Most patients with diffuse MYB/MYBL1-altered astrocytoma had epileptic seizures since childhood [15] and symptoms such as movement disorders, behavioral or mnestic changes occur with both encephalopathy and glioneuronal tumors [13, 14]. This symptom overlap complicates differentiation and may lead to overlooking encephalitis in individual cases. Conversely, not every MRI abnormality should be attributed to encephalitis, but should be carefully investigated even in patients with known NMDARE, as it may mask an underlying neoplasm.

Recently, two isolated cases of NMDARE and astrocytoma have been reported [1, 9]. Both studies speculated a link between tumor and encephalitis, but did not perform further analyses. While astrocytes do indeed express NMDARs, this is rarely the case in astrocytomas. Notably, RNA-seq data here show no expression of NR1 [3], the major target of autoantibodies in NMDARE [6]. Together with the high prevalence of common astrocytomas [10], it remains unclear whether these tumors are indeed associated with NMDARE or whether this is coincidence.

This study suggests a link between certain brain tumors and NMDARE. We show atypical NMDAR expression on dysmorphic neurons that may have prompted antibody formation and subsequent autoimmune encephalitis. Tumor resection led to a decrease in these antibodies and terminated a previously refractory encephalitis, supporting the association *ex juvantibus*. Thus, following findings on NMDARE in ovarian teratomas, we propose an etiologic concept in which, in addition to HSE, the immunogenic properties of dysmorphic neurons, whether outside or inside the brain, serve as triggers for NMDARE.

## Supplementary Information

Below is the link to the electronic supplementary material.

Supplementary file1 (DOCX 4247 KB)

## Data Availability

Data are available on request.
