# Supplementary material for: Atypical NMDA receptor expression in a diffuse astrocytoma, MYB- or MYBL1-altered as a trigger for autoimmune encephalitis
Source: Acta Neuropathol. 2022 Jun 21;144(2):385–9. doi: 10.1007/s00401-022-02447-y (PMC9288378; doi:10.1007/s00401-022-02447-y)
Supplement: Supplementary file 1 — Supplementary file1 (DOCX 4247 KB) [file 401_2022_2447_MOESM1_ESM.docx]

# *Acta Neuropathologica*

# **Atypical NMDA receptor expression in a diffuse astrocytoma, MYB- or MYBL1-altered as a trigger for autoimmune encephalitis**

*Marc Nikolaus, MD*; Arend Koch, MD*; Werner Stenzel, MD; Sefer Elezkurtaj, MD; Felix Sahm, MD; Anna Tietze, MD; Laura Stöffler, MD; Jakob Kreye, MD; Pablo Hernáiz Driever, MD; Ulrich W. Thomale, MD; Angela M. Kaindl, MD; Markus Schuelke, MD; Ellen Knierim, MD*

**Corresponding author:** Ellen Knierim, MD, Department of Neuropediatrics, Charité Universitätsmedizin Berlin, Augustenburger Platz 1, Mittelallee 8, 13353 Berlin; Phone +49 30 450 566112; Fax +49 30 450 566920; Email: ellen.knierim@charite.de

**Supplemententary 1: The case in detail**

A 21-month-old previously healthy girl presented with a four-week history of gait disturbance and severe behavioral changes including insomnia, agitation, and autoaggression. Two episodes of afebrile generalized tonic-clonic seizures were observed the night before admission. Physical examination revealed severe ataxia with inability to walk unassisted and inadequate speech with perseveration and delayed response, which later changed to mutism. There were abrasions on both hands, bite wounds on the lips, and missing hair due to continued self-mutilation. The girl's mRS score was 4 and NEOS score was 3. Blood chemistry and inflammatory markers were normal. An EEG showed two focal seizures with loss of consciousness, one-minute movement arrest, and epileptiform discharges in the right frontal lobe. The seizures were treated with levetiracetam (60 mg/kg/d). Neurologic examination on the same day revealed T_2_-white matter hyperintensity in the right cerebellar hemisphere without hemorrhage or calcification, minimal contrast uptake, and unremarkable MR spectroscopy and diffusion-weighted imaging, suggestive of an inflammatory lesion. CSF analysis showed mild lymphocytic pleocytosis (10 cells/µl), type 2 oligoclonal bands, and intrathecal antibody synthesis, whereas glucose, lactate, and protein levels were within normal ranges, and the search for foci of infection remained negative. Screening for antineuronal and paraneoplastic antibodies revealed the presence of anti-NMDAR IgG antibodies with high CSF (1:1,000) and serum titers (> 1:10,000). No tumor was found on chest radiography and abdominal ultrasonography. On the third day after admission, one month post symptom onset (pso), we diagnosed NMDARE due to the combination of encephalopathy with seizures, behavioral and movement disorders, abnormal T_2_-hyperintensity on MRI, and high-titer anti-NMDAR antibodies (full diagnostic results in **Supplementary Table 1**). Because 5 days of treatment with high-dose intravenous methylprednisolone (IVMP, 20 mg/kg/d) starting on the fourth day did not result in clinical improvement, immunotherapy was intensified by plasmapheresis (3 courses) and immunoadsorption (3 courses). Oral prednisolone (1 mg/kg/d) was introduced for long-term prophylaxis. In the following 14 days, the encephalopathy improved markedly, autoaggression with self-injurious behavior subsided, walking ability improved, and speech became possible again. Finally, the girl was discharged 2 months pso (mRS=1). A follow-up one month later revealed further improvement without seizures or motor deficits, but persistent nocturnal restlessness and daytime aggression. While serum anti-NMDAR antibodies remained high (titer >1:10,000), CSF analysis showed a normal cell count and a decrease in anti-NMDAR antibodies (titer 1:32). EEG was normal, but MRI still showed T_2_-hyperintensity of the cerebellum with a slight increase in size, now suggestive of a mass rather than mere inflammation. In view of these findings, both increased immunosuppression and PET MRI were recommended but not performed because of lack of compliance, and oral prednisolone and levetiracetam were continued unchanged for 10 months. A first relapse occurred 13 months pso with agitation and speech disturbance (mRS=2). Serum anti-NMDAR antibodies were still positive (titer 1:1,000), MRI showed no signs of inflammation but a slightly growing cerebellar mass without avidity on F18-FDG-PET analysis. Because of suspicion of brain tumor, FET-PET MRI and biopsy in conjunction with further lumbar puncture and IVMP were recommended but rejected by the parents. Fifteen months pso, the girl presented with a second relapse that included nocturnal agitation, other- and autoaggression, speech delay, and severe emotional instability (mRS=3). This time, lumbar puncture revealed type 3 oligoclonal bands, intrathecal antibody synthesis, and rising anti-NMDAR antibodies (titer 1:100) consistent with their rise in serum (titer 1:10,000). After another five-day treatment with IVMP, symptoms resolved over the following month (mRS = 1 to 2), whereas oral corticosteroids and levetiracetam were slowly discontinued. FET-PET MRI confirmed a progressive cerebellar mass with low metabolic signal, suggestive of glioma. At 16 months pso, tumor tissue was obtained during a navigated needle biopsy. Based on histomorphology and molecular profile, diffuse astrocytoma, MYB- or MYBL1-altered with a MYBL1:MMP16 fusion was detected. Because of the localization without obstruction of the ventricular system and the patient's clinical improvement combined with a decrease in anti-NMDAR antibodies (titer 1:32), we decided to adopt a wait-and-see strategy. However, 23 months later, a third relapse occurred with increased insomnia, nocturnal restlessness, and behavioral abnormalities, as well as an apparent delay in speech development (mRS = 3). CSF analysis again revealed pleocytosis and rising anti-NMDAR antibodies (titer 1:100). Cranial MRI showed persistent slow progression of the low-grade glial tumor. After further immunoadsorption (6 courses), we proposed neurosurgical tumor debulking to achieve a beneficial effect on tumor progression and NMDARE, similar to that observed in cases with ovarian teratomas. Finally, 27 months pso, R2 resection of the cerebellar tumor was successful without complications. After 4 weeks of rehabilitation, the girl did not show motor deficits anymore, while insomnia and autoaggression had disappeared, and delayed speech improved. At the last follow-up at 33 months pso, MRI showed a stable remaining tumor mass, and CSF analysis revealed that anti-NMDAR antibodies were at an all-time low (titer 1:3). Examination (mRS = 1) and neurocognitive assessment confirmed clinical restitution.

**Table S1** Detailed diagnostic results

| Test | from admission to diagnosis (1 month pso) |
| --- | --- |
| Routine blood test | ***Normal:*** electrolytes, blood count, parameters for hemolysis, liver/kidney function, thyroid hormones, inflammation, and coagulation parameters. |
| Serological  analysis | ***Normal/negative IgM/IgG for:*** CMV, EBV, enterovirus, hepatitis A/B/C, HIV, HHV6, HSV1/2, JC virus, parechovirus and varicella, *Borrelia burgdorferi, Haemophilus influenzae, Listeria monocytogenes, Neisseria meningitidis, Streptococcus agalactiae, Streptoccocus pneumoniae,* cryptococcosis, toxoplasmosis, and leptospirosis. |
| Immunology | ***Normal:*** immunoglobulins, immunoglobulin subclasses, antibodies to vaccination (anti-measles, -tetanus, aPCP-IgG), distribution of leukocytes and subpopulations within normal range; |
| CSF analysis | ***Pathologic:*** 10 cells/µl [<5], oligoclonal bands (OCB) positive in CSF, negative in serum (type 2), CSF IgG index > 0.7.  ***Normal:*** glucose 55 mg/dl, protein 151 mg/dl, lactate 12.9 mg/dl |
| Anti-neuronal  antibodies | ***Pathologic:* Serum**: anti-NMDAR > 1:10,000↑↑↑ [negative] for CBA. **CSF**: anti-NMDAR 1:1000↑↑ [negative] for CBA.  ***Normal:* Serum** and **CSF**: anti-neuronal IgG panel ("Biochip Mosaic", EUROIMMUN Clinical Immunology Laboratory, Lübeck, Germany) including Hu, Ro, ANNA-3, Yo, Tr/DNER, myelin, Ma/Ta, GAD65, anti-amphiphysin, anti-aquaporin-4, anti-NMDA, AMPA, GABA_B_-R, LGl1, ZIC4, CASPR2, DPPX, Glycine-R, mGluR1, mGluR5, ARHGAP26, ITPR1, CARPVIII, Homer3, MOG, Recoverin, Neurochondrin, GluRD2, Flotillin, IgLON5) in a commercial cell-based assay (CBA). |
| Electro-  physiology | ***Pathologic:*** EEG with epileptiform discharges and two focal seizures of the right frontal lobe, with subtle clinical correlation of motor arrest and unresponsiveness for up to 30 seconds. |
| Imaging | ***Pathologic:*** cerebral MRI, MR spectroscopy with T2 hyperintense signal change, right-sided in cerebellar white matter, with no evidence of hemorrhage or calcification, almost no contrast uptake, and unremarkable signatures on MR spectroscopy and diffusion-weighted sequences.  ***Normal:*** chest X-ray, abdominal ultrasonography, ECG. |

Laboratory tests were performed in the clinical laboratory affiliated with Charité (Labor Berlin, Charité Vivantes GmbH, Berlin); normal values/ranges are indicated in square brackets [ ].

**Supplementary 2: Methods**

**Standard protocol approvals, registrations and study recruitment**

The ethics committee of Charité-Universitätsmedizin Berlin approved this study (EA2/121/17). Written informed consent was obtained from the patients or their legal guardians for the storage and use of the samples and clinical information for research purposes. All mouse experiments were performed in accordance with local animal welfare regulations (LaGeSo Berlin registration number T0118/17).

**Immunological studies with patient CSF on mouse brain tissue and in cell culture.**

Tissue-based assay (TBA) was performed according to established protocols [5]. For the cell-based assay (CBA), HEK293T cells were transiently transfected with NR1 DNA (1 µg). CSF staining and confocal microscopy were performed as previously described [4].

**Immunological investigations on tumor tissue from patients and controls**

A tissue sample was obtained from the patient's tumor biopsy. In addition, we examined tissues from two healthy controls, two cases of NMDARE without brain tumor, two cases of *Herpes simplex* encephalitis (HSE), and three different low-grade brain tumor entities-two pilocytic astrocytomas (PA) located at a lateral and the 4^th^ ventricle, six gangliogliomas (GG), all from temporal or frontal lobe areas, and two supratentorial dysembryoplastic neuroepithelial (DNT) tumors. Staining was performed on 3 μm tissue sections from formalin-fixed, paraffin-embedded, and fresh-frozen biopsy material using an automated slide staining system (BenchMark XT, Ventana Medical Systems, Tucson, AZ) according to established protocols including appropriate positive and negative control staining procedures. Heat-induced epitope retrieval (Cell Conditioning 1, Ventana) was used for antigen retrieval. For automated staining with secondary antibodies, the DAB detection kit (IVIEW, Ventana Medical Systems, Tucson, AZ) was used. H&E staining was performed according to standard protocols. Details of the antibodies are given in **Table S2.**

**Molecular and epigenetic characterization of the patient's tumor**

For genome-wide DNA methylation profiling analysis, DNA was isolated from a paraffin-embedded tissue sample and processed using an Illumina EPIC array (850k). The methylation pattern was matched to over 2,800 reference cases from 82 brain tumor entities using a DNA methylation-based classification algorithm (www.molecularNeuropathology.org; Classifier version V11b4 and V12). Agreement with an established methylation class was verified for a calibrated classifier score cut-off of 0.9. In pediatric cases, an approximation to this threshold was suggested to be sufficient for matching because of the smaller tumor mass [1, 2]. RNA-Seq data can be used to detect expressed fusion genes. RNA sequencing was performed using next generation sequencing (Illumina TruSeq RNA Access; sequencing on Illumina NextSeq). [3]. Raw data were then aligned, and gene fusion was identified using Arriba (v1.1.0).

**Table S2** Immunological studies on tumor tissue from patients and controls

| Antibody | Host/target | Source | Clone | Dilution | Cells stained |
| --- | --- | --- | --- | --- | --- |
| CD3 | mouse anti-human | Dako | UCHT1 | 1:100 | T lymphocytes |
| CD8 | mouse anti-human | Dako | EBM1 | 1:100 | CD8+ T cells |
| CD45 | mouse anti-human | Dako | 2B11+PD7/26 | 1:400 | Leukocytes |
| Synaptophysin | mouse anti-human | Synaptic Systems | 7.2 | 1:250 | Neuron terminals |
| MAP2 | mouse anti-human | Sigma | HM-2 | 1:15,000 | Neuron dendrites, oligodendrocytes |
| KI67 | mouse anti-human | Dako | MIB-1 | 1:100 | Proliferating cells |
| NR1 | mouse anti-human | Sigma | 54.1 | 1:250 | NMDAR subunit 1, neurons, ganglion cells |
| NR2b | mouse anti-human | Invitrogen | B3-13B11 | 1:100 | NMDAR subunit 2b |
| GFAP | rabbit anti-human | Dako | polyclonal | 1:2,000 | Astrocytes |

**Supplementary 3: Ganglioglioma**

**
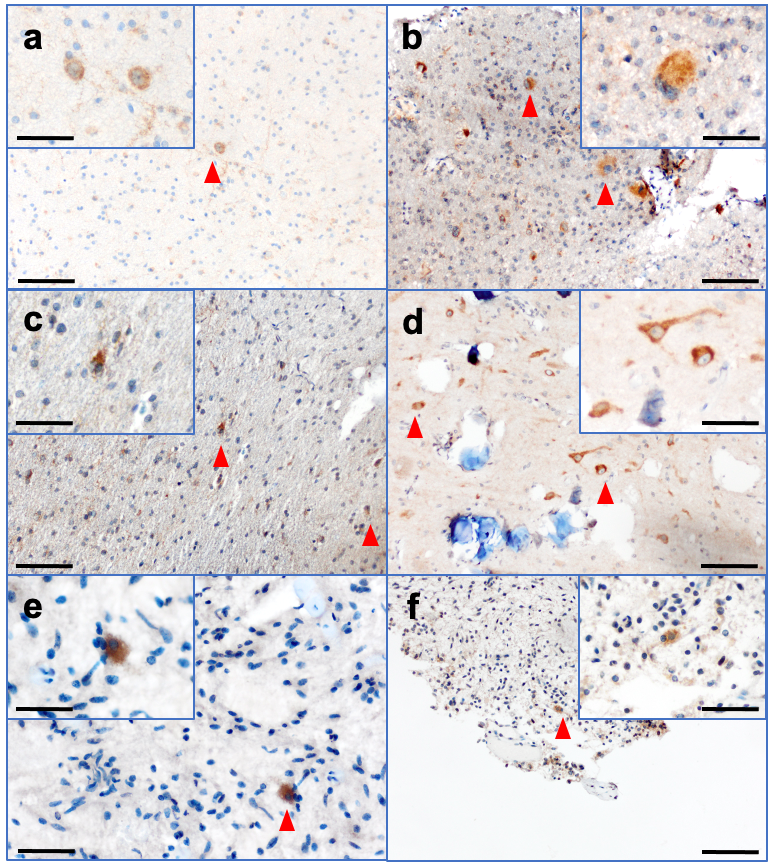
**

Immunostaining of formalin-fixed, paraffin-embedded biopsy specimens from six ganglioglioma patients. All six reproducibly showed neuropil staining depending on their neuronal and glial tissue composition and NR1-positive dysplastic neurons (red arrowheads) with the atypically expressed immunoreactivity focused on cell bodies. GG = ganglioglioma. Size bars: 200 µm (a-f), 100 µm (insets)

**Supplementary 4: NMDARE-associated Ovarian teratoma**


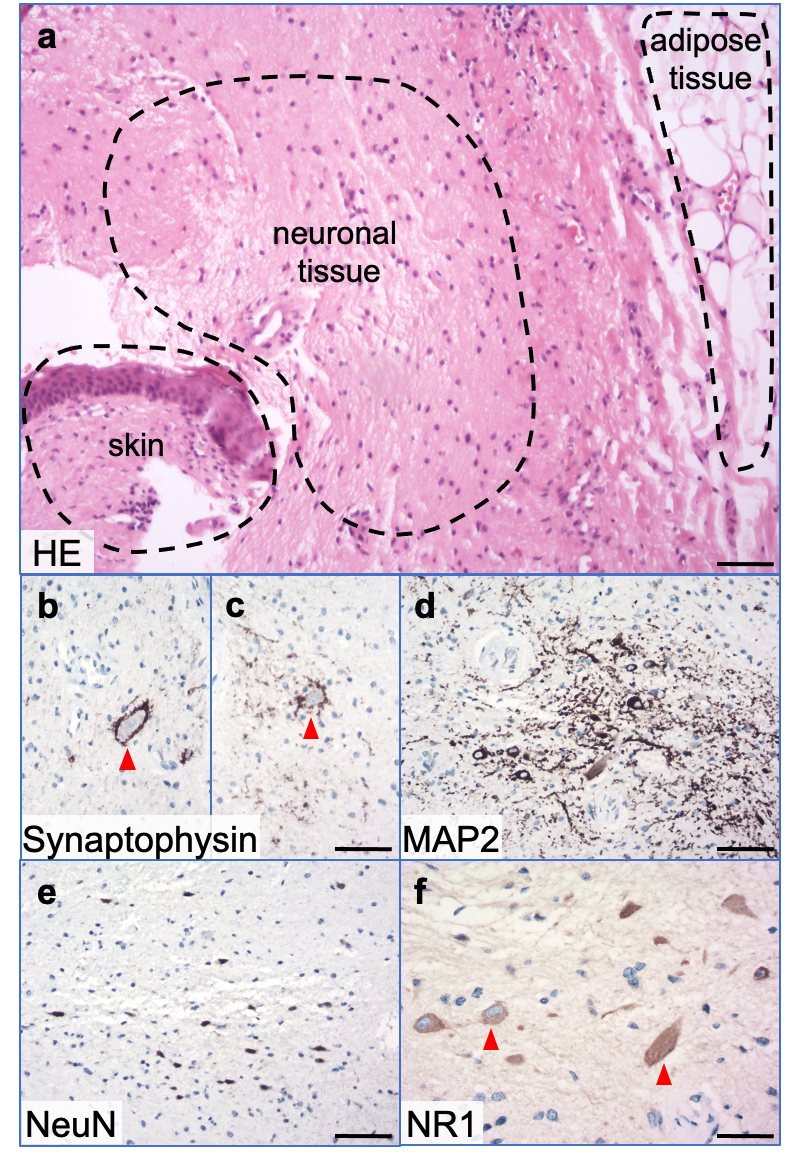


Immunostaining of formalin-fixed, paraffin-embedded biopsy from NMDARE-associated ovarian teratoma (a) and characterization of the nervous tissue it contains (b-f). Dysmorphic neurons display perisomatic synaptophysin staining (arrowheads, b, c). MAP2-positive processes show neurogial components (d). Loss of NeuN indicates altered neurons (e). Dysmorphic neurons (arrowheads) show NMDAR-positivity with atypical concentration in somata rather than in neuropil. Size bars: 200 µm (a-e), 100 µm (f).

**Supplementary 5: Deep cerebellar nuclei**


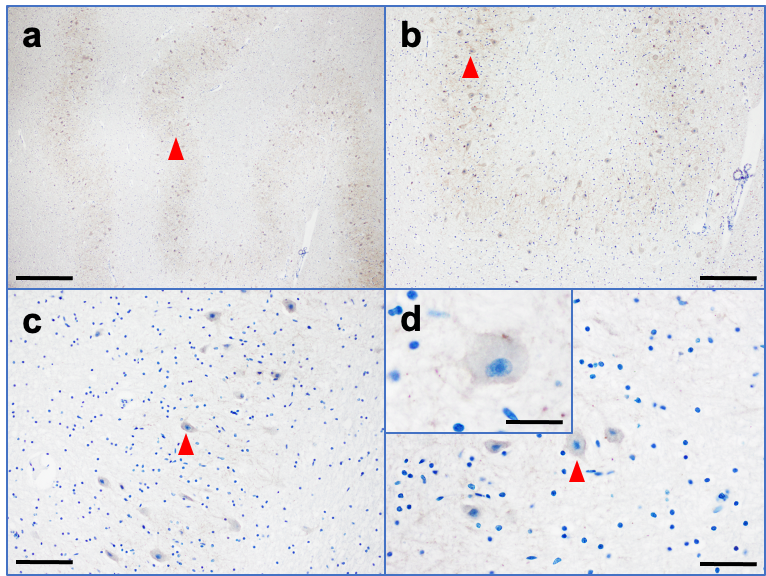


Immunostaining of formalin-fixed, paraffin-embedded cerebellar biopsy from NMDARE without brain tumor or viral infection. Overview (a, b) and detailed (c, d) images of the dentate nucleus within the deep cerebellar nuclei (DCN). Anti-NR1 staining displays the subtle neuropil signal (a, arrowhead) and both border and central neurons with NMDAR-negative somata (c, d, inset, arrowheads). Size bars: 200 µm (a-d), 100 µm (inset).

**Supplemental References**

1. Capper D, Jones DTW, Sill M, Hovestadt V, Schrimpf D, Sturm D, Koelsche C, Sahm F, Chavez L, Reuss DE, Kratz A, Wefers AK, Huang K, Pajtler KW, Schweizer L, Stichel D, Olar A, Engel NW, Lindenberg K, Harter PN, Braczynski AK, Plate KH, Dohmen H, Garvalov BK, Coras R, Hölsken A, Hewer E, Bewerunge-Hudler M, Schick M, Fischer R, Beschorner R, Schittenhelm J, Staszewski O, Wani K, Varlet P, Pages M, Temming P, Lohmann D, Selt F, Witt H, Milde T, Witt O, Aronica E, Giangaspero F, Rushing E, Scheurlen W, Geisenberger C, Rodriguez FJ, Becker A, Preusser M, Haberler C, Bjerkvig R, Cryan J, Farrell M, Deckert M, Hench J, Frank S, Serrano J, Kannan K, Tsirigos A, Brück W, Hofer S, Brehmer S, Seiz-Rosenhagen M, Hänggi D, Hans V, Rozsnoki S, Hansford JR, Kohlhof P, Kristensen BW, Lechner M, Lopes B, Mawrin C, Ketter R, Kulozik A, Khatib Z, Heppner F, Koch A, Jouvet A, Keohane C, Mühleisen H, Mueller W, Pohl U, Prinz M, Benner A, Zapatka M, Gottardo NG, Driever PH, Kramm CM, Müller HL, Rutkowski S, Hoff K von, Frühwald MC, Gnekow A, Fleischhack G, Tippelt S, Calaminus G, Monoranu C-M, Perry A, Jones C, Jacques TS, Radlwimmer B, Gessi M, Pietsch T, Schramm J, Schackert G, Westphal M, Reifenberger G, Wesseling P, Weller M, Collins VP, Blümcke I, Bendszus M, Debus J, Huang A, Jabado N, Northcott PA, Paulus W, Gajjar A, Robinson GW, Taylor MD, Jaunmuktane Z, Ryzhova M, Platten M, Unterberg A, Wick W, Karajannis MA, Mittelbronn M, Acker T, Hartmann C, Aldape K, Schüller U, Buslei R, Lichter P, Kool M, Herold-Mende C, Ellison DW, Hasselblatt M, Snuderl M, Brandner S, Korshunov A, Deimling A von, Pfister SM (2018) DNA methylation-based classification of central nervous system tumours. Nature 555:469–474. doi: 10.1038/nature26000

2. Capper D, Stichel D, Sahm F, Jones DTW, Schrimpf D, Sill M, Schmid S, Hovestadt V, Reuss DE, Koelsche C, Reinhardt A, Wefers AK, Huang K, Sievers P, Ebrahimi A, Schöler A, Teichmann D, Koch A, Hänggi D, Unterberg A, Platten M, Wick W, Witt O, Milde T, Korshunov A, Pfister SM, Deimling A von (2018) Practical implementation of DNA methylation and copy-number-based CNS tumor diagnostics: the Heidelberg experience. Acta Neuropathol 136:181–210. doi: 10.1007/s00401-018-1879-y

3. Jones DTW, Hutter B, Jäger N, Korshunov A, Kool M, Warnatz H-J, Zichner T, Lambert SR, Ryzhova M, Quang DAK, Fontebasso AM, Stütz AM, Hutter S, Zuckermann M, Sturm D, Gronych J, Lasitschka B, Schmidt S, Seker-Cin H, Witt H, Sultan M, Ralser M, Northcott PA, Hovestadt V, Bender S, Pfaff E, Stark S, Faury D, Schwartzentruber J, Majewski J, Weber UD, Zapatka M, Raeder B, Schlesner M, Worth CL, Bartholomae CC, Kalle C von, Imbusch CD, Radomski S, Lawerenz C, Sluis P van, Koster J, Volckmann R, Versteeg R, Lehrach H, Monoranu C, Winkler B, Unterberg A, Herold-Mende C, Milde T, Kulozik AE, Ebinger M, Schuhmann MU, Cho Y-J, Pomeroy SL, Deimling A von, Witt O, Taylor MD, Wolf S, Karajannis MA, Eberhart CG, Scheurlen W, Hasselblatt M, Ligon KL, Kieran MW, Korbel JO, Yaspo M-L, Brors B, Felsberg J, Reifenberger G, Collins VP, Jabado N, Eils R, Lichter P, Pfister SM, Project ICGCPT (2013) Recurrent somatic alterations of FGFR1 and NTRK2 in pilocytic astrocytoma. Nat Genet 45:927–932. doi: 10.1038/ng.2682

4. Kreye J, Wenke NK, Chayka M, Leubner J, Murugan R, Maier N, Jurek B, Ly L-T, Brandl D, Rost BR, Stumpf A, Schulz P, Radbruch H, Hauser AE, Pache F, Meisel A, Harms L, Paul F, Dirnagl U, Garner C, Schmitz D, Wardemann H, Prüss H (2016) Human cerebrospinal fluid monoclonal N-methyl-D-aspartate receptor autoantibodies are sufficient for encephalitis pathogenesis. Brain 139:2641–2652. doi: 10.1093/brain/aww208

5. Nikolaus M, Meisel C, Kreye J, Prüss H, Reindl M, Kaindl AM, Schuelke M, Knierim E (2020) Presence of anti-neuronal antibodies in children with neurological disorders beyond encephalitis. Eur J Paediatr Neuro 28:159–166. doi: 10.1016/j.ejpn.2020.07.004
